# Announcement

**Published:** 2015-07-03

## National Cleft and Craniofacial Awareness and Prevention Month — July 2015

July is National Cleft and Craniofacial Awareness and Prevention Month, a time to raise awareness and improve understanding of orofacial clefts (clefts of the lip and palate) and other conditions of the head and face. Each year in the United States, approximately 2,600 babies are born with a cleft palate and 4,400 babies are born with a cleft lip, with or without a cleft palate (1). Other craniofacial birth defects include craniosynostosis (skull sutures fusing prematurely), anotia/microtia (ear is missing or underdeveloped), and anophthalmia/microphthalmia (missing or abnormally small eye).

Children with orofacial clefts and other craniofacial conditions often have impaired ability to feed and impaired language development, and might be at increased risk for a greater number of ear infections, hearing issues, and problems with their teeth. Because of the high prevalence of orofacial clefts and health care use and costs associated with treatment, improving the health of these children is an important public health goal (2). CDC and its partners are working to better understand the preventable causes of clefts and craniofacial defects, and how these conditions affect children and their families, by focusing on risk factors, health care–service use, access to care, quality of life, health outcomes, and management and treatment of these conditions.

To help reduce a woman’s risk for having a baby with an orofacial cleft or other craniofacial condition, health care providers should encourage patients who are thinking about becoming pregnant to commit to a healthy lifestyle (e.g., control diabetes, quit smoking) before becoming pregnant. Health care providers should also work with them to make informed decisions about medication treatment during pregnancy. Additional information regarding National Cleft and Craniofacial Awareness and Prevention Month is available at http://www.nccapm.org/about.html.
